# Sick Headache

**Published:** 1873-10

**Authors:** 


### SICK HEADACHE.

The true cause of sick headache lies deep in the patient's idiosyncrasy, and is developed by a hundred different causes. The advice, then, to sufferers is to give as much tone as they can to their nerves by adopting all those methods which experience has shown to be good, and then avoid, as far as practicable, all those causes which are known to excite an attack. I need scarcely describe a sick headache—how one rises in the morning more dead than alive; perfectly unable to swallow the smallest particle of food, and often, perhaps, actually sick; how the head throbs, and the pain increased by the slightest movement; how speaking or doing is a burden beyond bearing; how one prays to be left alone in the utmost quiet, so that he may, if possible, sleep. To other persons the sufferer looks extremely ill, very pale, dark around the eyes, and with contracted pupil. To himself his head feels hot, and the application of cold is most refreshing. The clamminess in the mouth, the nausea and general gastric disturbances are secondary, and have no connection with any improper meal, and thus is in no way relieved by the too frequent and ignorantly administered purgative. This is not needed, and has no good result.—The only remedies which are of any avail are those which act on the nervous system, such as hot tea and coffee; or, after the stomach is quieter, and the more urgent symptoms have passed off, a little wine or ammonia. If the headache take more the form of hemicrania, then remedies are occasionally useful, as the local application of the bisulphide of carbon, or galvanism, and internally the bromide of potassium. This is the only drug which I have really seen to be serviceable. Whilst the nausea, exists and the worst symptoms prevail, even this remedy is of no avail.—*British Medical Journal.*

The reason why prawn, rock fish, oysters, etc., are, at certain seasons, unwholesome articles of food, and even poisonous, it is said, depends upon a change of blood connected with the process of spawning.

